# Validation of the Arabic version of the “self-evaluation of negative symptoms” scale (SNS)

**DOI:** 10.1186/s12888-020-02647-4

**Published:** 2020-05-14

**Authors:** Aline Hajj, Souheil Hallit, Karam Chamoun, Hala Sacre, Sahar Obeid, Chadia Haddad, Sonia Dollfus, Lydia Rabbaa Khabbaz

**Affiliations:** 1 Faculty of Pharmacy, Saint-Joseph University, Beirut, Lebanon; 2 Laboratoire de Pharmacologie, Pharmacie Clinique et Contrôle de Qualité des Médicaments, Saint-Joseph University, Beirut, Lebanon; 3 Faculty of Medicine and Medical Sciences, Holy Spirit University of Kaslik (USEK), Jounieh, Lebanon; 4 INSPECT-LB: Institut National de Santé Publique, Epidémiologie Clinique et Toxicologie, Beirut, Lebanon; 5 Drug Information Center, Order of Pharmacists of Lebanon, Beirut, Lebanon; 6 Research Department, Psychiatric Hospital of the Cross, P.O Box 60096, Jal Eddib, Lebanon; 7 Faculty of Arts and Sciences, Holy Spirit University of Kaslik (USEK), Jounieh, Lebanon; 8 CHU de Caen, Service de Psychiatrie, 14000 Caen, France; 9 Normandie Univ, UNICAEN, ISTS, GIP Cyceron, 14000 Caen, France

**Keywords:** Arabic, Negative symptoms, Schizophrenia, Self-evaluation of negative symptoms scale, SNS, Validation

## Abstract

**Background:**

The self-evaluation of negative symptoms scale (SNS) is a new easy-to-use self-administered questionnaire allowing clinicians to understand the clinical and genetic factors affecting the negative symptoms in patients with schizophrenia. There was a need to translate and validate this scale in Arabic so that Arab-speaking patients benefit from it. Therefore, the aim of our study was to validate the Arabic version of the SNS in a sample of Lebanese patients with schizophrenia.

**Methods:**

The Arabic SNS was used to quantify the disability associated with negative symptoms in patients with schizophrenia (*n* = 206). Six weeks after completing the SNS, the participants were interviewed again to assess test-retest reproducibility. The validity was confirmed by factor analyses using the principal component analysis technique with a varimax rotation. The Positive and Negative Syndrome Scale (PANSS) was also assessed.

**Results:**

None of the items of the SNS scale were removed; all items converged over a solution of five factors that had an eigenvalue > 1, explaining a total of 66.01% of the variance (Cronbach’s alpha = 0.879; test part). The mean total SNS score was 17.33 ± 8.43 for the “test”, and 16.35 ± 7.50 for the “retest”. The correlation coefficients between the SNS total score and the PANSS scale and subscales were as follows: total PANSS (r = 0.044; *p* = 0.530), positive PANSS score (r = − 0.106; *p* = 0.131), negative PANSS score (r = 0.204; *p* = 0.003), and general psychopathological PANSS score (r = 0.03; p = 0.530).

**Conclusion:**

This study is the first to validate the Arabic version of the SNS in patients with schizophrenia. Using this scale would help improve treatment by correctly assessing negative symptoms, thus optimizing treatment options.

## Background

Schizophrenia is a severe and complex neurodevelopmental disorder, and one of the most disabling medical conditions contributing to the global burden of disease [1]. Its heterogeneity in the clinical expression and symptoms of patients leads to high rates of resistance to treatment and lower quality of life [2, 3]. Studies have shown that individuals with schizophrenia exhibit a mosaic of symptoms encompassing positive psychotic symptoms, severe cognitive impairment (such as, low levels of reasoning, decreased speed of execution, etc.), and, most importantly, negative symptoms that include several domains ranging from alogia, avolition, anhedonia, social withdrawal, and blunted affect, and can widely vary between patients; it is even considered as strong predictors of treatment outcome and response [4, 5]. Indeed, when poorly assessed, negative symptoms could lead to worse outcomes of global disability, with lack of psychological adjustment and emotional relationship, low levels of achievement in everyday tasks, impaired community functioning, and much lower rates of remission over time [6]. Moreover, evaluating these symptoms in schizophrenia is challenging as it requires the use of standardized evaluation tools for a reliable assessment [7, 8]. Hence, the importance of correctly assessing and identifying negative symptoms and understanding correlates to help health professionals develop an adequate treatment strategy for each patient.

Several tools were designed primarily to measure symptoms in patients with schizophrenia, most of which relied on hetero-assessments, of which the Positive and Negative Syndrome Scale (PANSS) that measures both negative and positive symptoms of patients. However, recent studies highlighted the importance of subjective assessment by schizophrenic patients [9], since it allows them to assess their overall functioning, and requires their participation in analyzing their symptoms. This can be considered as a basis for cognitive/social therapy of patients with schizophrenia [6], especially in the early stages of the disease. However, several limitations arise when using subjective assessment scales of negative symptoms, including non-specificity, and the lack of items addressing the five negative dimensions of deficit [10]. The self-evaluation of negative symptoms scale (SNS) is a new easy-to-use self-administered questionnaire, covering the five sub-domains of negative symptoms, and allowing patients to express their deficits in motivation and speech expression as well as their loss of emotion regardless of their depressive symptoms [10–12]. Indeed, in Dollfus et al. study [10], alogia and avolition/apathy subdomains of the SNS were significantly correlated with the corresponding SANS subscales whereas no correlation was observed between the emotional range of the SNS and depressed mood [10–12]. It has been translated into more than 16 different languages [6], but not in Arabic, thus the need to translate and validate the SNS in Arabic so that Lebanese and Arab-speaking patients benefit from this tool.

Therefore, the aim of this study was to validate the Arabic version of the SNS and compare it with the negative symptoms subscale of the PANSS in a sample of Lebanese patients with schizophrenia.

## Methods

### Study design

A prospective clinical study was conducted from October 2018 to April 2019 at the Psychiatric Hospital of the Cross (HPC, Jal El Dib, Metn, Lebanon). The database of in-patients with schizophrenia identified 306 eligible patients, of whom 206 were randomly selected using an online software (www.randomizer.org). Inclusion criteria were: age more than 18 years, a primary diagnosis of schizophrenia by a physician according to the Diagnostic and Statistical Manual 5th Edition (DSM-5) criteria, duration of hospitalization of more than a year (to be classified as chronic patients), clinical stability on current treatment without significant change in psychotropic drugs/doses for three months or more. Patients not included were those hospitalized for less than a year, with variable medication doses, who had autism or bipolar disorder or personality disorder or a severe organic disorder, including central nervous system disorders that may affect the cognitive function (dementia, multiple sclerosis, epilepsy, Parkinson’s disease, and mental retardation), and those who refused to participate in the study. Participants received no financial incentive. The 206 selected patients were included in the validation of the SNS scale. The methodology used was also described elsewhere [REFERENCES: Hajj A, Obeid S, Sahyoun S, et al. Clinical and Genetic Factors Associated with Resistance to Treatment in Patients with Schizophrenia: A Case-Control Study. Int J Mol Sci. 2019;20(19). AND Dahdouh O, Haddad C, Hany Z, et al. Colour discrimination among patients with schizophrenia in Lebanon. Int J Psychiatry Clin Pract. 2020:1-8. AND Haddad C, Darwich MJ, Obeid S, et al. Factors associated with anxiety disorders among patients with substance use disorders in Lebanon: Results of a cross-sectional study. Perspect Psychiatr Care. 2019.

### Ethical aspect

The study was approved by ethical committees of Hôtel-Dieu de France Hospital (CEHDF1017) and the HPC (HPC-017-2017). Participants were fully informed of the purpose and procedures of the study and had the adequate time to ask questions and ponder about their voluntary participation. All patients or their legal representatives gave their written informed consent prior to inclusion.

### Sample size calculation

Comrey and Lee suggested that a minimum of 10 observations per variable is necessary to perform an exploratory factor analysis [13]. Since the SNS is a 20-item questionnaire, a minimum of 200 patients was required for this study.

### Sociodemographic information

Clinical and demographic information included age, gender, ethnicity/nationality, marital status, education level, and the use of alcohol, tobacco and other psychoactive substances. All psychiatric details (related or not to schizophrenia) were recorded: family/personal history of schizophrenic episodes, family/personal history of psychiatric disorders, time since the diagnosis of schizophrenia, number of episodes, start date of the actual episode, time since the start of antipsychotic treatment for the actual episode, anti-psychotic treatment (dose per 24 h as given daily by the nurses at HPC), other medications. The chlorpromazine-equivalent daily dose of typical and atypical antipsychotics was calculated according to published guidelines [14].

### SNS validation

After obtaining the approval from Professor Sonia Dollfus, who holds the copyright of the scale, the SNS was translated from French into Arabic through an initial translation and then verified through a back-translation process (each time by independent professional translators). The research team and the translators then compared the French versions of the SNS to determine whether the questions had the same meaning. Corrections were made when necessary (Certificate of linguistic validation, September 10, 2018). The final version included 20 items with a scoring sheet.

The questionnaire was administered twice (noted Test and Retest) to validate the Arabic version of the SNS scale. The interval between the two appointments was 6 weeks, during which patients were expected to exhibit stability in symptoms and treatment [10]. Patient filled-out the questionnaire, which required less than 5 min to complete, in the presence of a specialized clinical psychologist for further explanations/specific help. The questionnaire was easy to understand and complete, and responses were scored on a 3-point Likert scale: 0 “strongly disagree”, 1 “somewhat agree”, and 2 “strongly agree”. Five partial results or sub-scores were obtained corresponding to five distinct categories of negative symptoms: 1) Social withdrawal (Items 1–2–3-4 assessing social and familial relationships, friendships, and the patient’s desire to establish new relationships); 2) Diminished emotional range or emotional withdrawal (Items 5–6–7-8 assessing perceived happiness or sadness in relatively happy or sad situations; 3) Alogia (Items 9–10–11-12 assessing the effort required by the patient to speak); 4) Avolition (Items: 13–14–15-16 assessing the difficulty of reaching goals set in everyday life, desire, motivation, and energy); 5) Anhedonia (Items 17–18–19-20 assessing the perceived pleasure with the entourage, and the consummative and anticipatory pleasure. These sub-scores are summed to obtain a total score ranging from 0 (no negative symptoms) to 40 (severe negative symptoms). A trained research assistant performed data collection and made sure that all questions were answered.

### PANSS assessment

The Arabic version [15] of the PANSS was used to measure the negative symptoms of patients and compare them with those obtained by the SNS. This 30-item tool consists of 3 sub-scales: 7 for the positive scale, 7 for the negative scale, and 16 for the general psychopathological scale. The total score is calculated by summing all the answers [16]. The Cronbach’s alpha values were: total scale (α = 0.941), positive scale (α = 0.823), negative scale (α = 0.873), and psychopathological (α = 0.901).

### Statistical analysis

Statistical analysis was performed using SPSS software version 25.0. Descriptive statistics were calculated for all variables in the study. The normality of the variables within each group was verified by the Kolmogorov-Smirnov test. The Pearson’s correlation test was used to check for association between continuous variables. A *p* ≤ 0.05 was considered statistically significant. The validity of the construction of the SNS scale in this sample was confirmed by launching a factor analysis for the 20 items of the questionnaire, using the principal component analysis technique, with a varimax rotation since the extracted factors were not found to be significantly correlated. The Kaiser-Meyer-Olkin (KMO) measurement and the Bartlett sphericity test were performed to ensure the adequacy of the sampling. The number of factors retained corresponded to Eigen values greater than one. In addition, Cronbach’s alpha was recorded for reliability analysis of the total score and subscale factors: α ≥0.7 and ≥ 0.8 were considered as acceptable and excellent internal consistency values respectively [17]. The “test-retest” reliability was evaluated by the intra-class correlation coefficient (ICC, mean measurement) for the scores of the scales. Values less than 0.5, between 0.5 and 0.75, between 0.75 and 0.9, and greater than 0.90 were indicative of poor, moderate, good, and excellent reliability, respectively [18].

## Results

A total of 206 patients were included in this study. The mean age of the patients was 52.68 ± 12 years, with 56.8% women (*n* = 117), 81.6% (*n* = 168) single, 13.6% (*n* = 28) married, 4.4% (*n* = 9) divorced, and 62.1% (*n* = 128) had a primary education level. Moreover, 4.9% (*n* = 10) of patients declared having used psychoactive substances (mainly cannabis, cocaine, hashish), 7.8% (n = 16) of patients used alcohol, and 55.8% (*n* = 115) were smokers.

With respect to family history, 21.4% (*n* = 44) of patients had a family history of schizophrenic episodes, and 29.5% (*n* = 26) had a family history of other psychiatric/neurological conditions, including dementia, paranoia, personality disorders, mental retardation, depression, bipolar disorders, autism spectrum disorders, and epilepsy.

The mean total equivalent dose of chlorpromazine among patients was 1150.91 ± 973.70 mg. The equivalent dose of chlorpromazine for typical antipsychotics was 1010.57 ± 973.63 mg, whereas that for atypical antipsychotics was 163.57 ± 305.97 mg (Table 1).

**Table 1:** Sociodemographic and other characteristics of the patients (*N* = 206)^a^

		Frequency (%)
Gender	Men	89 (43.2%)
	Women	117 (56.8%)
Marital status	Single	168 (82%)
	Married	28 (13.7%)
	Divorced	9 (4.3%)
Educational level	Primary	128 (63.4%)
	Secondary	57 (28.2%)
	University	17 (8.4%)
Intake of psychoactive substances	No	196 (95.1%)
	Yes	10 (4.9%)
Alcohol intake	No	189 (92.2%)
	Yes	16 (7.8%)
Smoking	No	90 (43.9%)
	Yes	115 (56.1%)
Presence of family history of schizophrenic episodes	No	162 (78.6%)
	Yes	44 (21.4%)
Presence of family history of other psychiatric diseases	No	185 (89.9%)
	Yes	21 (10.2%)
		**Mean ± Standard Deviation**
Age (in years)		52.68 ± 12.00
Chlorpromazine total equivalent dose (in mg)		1150.91 ± 973.70
Chlorpromazine equivalent dose for typical antipsychotics (in mg)		1010.57 ± 973.63
Chlorpromazine equivalent dose for atypical antipsychotics (in mg)		163.57 ± 305.97

^a^ Some variables did not sum up to 206 due to missing data

### Validation of the SNS scale

#### SNS test results (Table 2)

A factor analysis, using the Principal Component Analysis (PCA), was carried out using the Varimax rotation since the items were not highly correlated. The PCA of the SNS scale was run over the whole sample (Total *n* = 206). None of the SNS scale items was removed; items converged over a solution of five factors that had an Eigenvalue over 1, explaining a total of 66.01% of the variance (Table 2). A Kaiser-Meyer-Olkin measure of sampling adequacy of 0.815 was found, with a significant Bartlett’s test of sphericity (*p* < 0.001).

**Table 2:** Results of the Varimax rotated matrix of the SNS items (Test)

Factor	Item number	Diminished emotional range	Avolition	Alogia	Social withdrawal	Anhedonia
There are many happy or sad things in life but I don’t feel concerned by them	6	0.755				
Watching a sad or happy film, reading or listening to a sad or happy story does not especially make me want to cry or laugh	7	0.715				
It is difficult for people to know how I feel	8	0.659				
People say I’m not sad or happy and that I’m not often angry	5	0.647				
I don’t particularly try to contact and meet friends (letters, telephone, text messaging, etc.)	4	0.552				
With friends and family, I want to talk about things but it doesn’t come out	12	0.514				
It’s hard to stick to doing things on an everyday regular basis	14		0.855			
There are many things I don’t do through lack of motivation or because I don’t feel like it	15		0.789			
I know there are things I must do (get up or wash myself for example) but I have no energy	16		0.764			
I find it difficult to meet the objectives I set myself	13		0.589			
I find it 10 times harder to talk than most people do	10			0.818		
People often say that I don’t talk much	11			0.796		
I don’t have as much to talk about as most people	9			0.784		
I prefer to be alone in my corner	1				0.871	
I’m better off alone, because I feel uncomfortable when anyone is near me	2				0.847	
I’m not interested in going out with friends or family	3				0.614	
When I imagine doing one thing or another, I don’t feel any particular pleasure in the idea	19					0.876
I find it hard to take pleasure even when doing things I have chosen to do	18					0.873
I am not interested in having sex	20					0.450
I don’t take any great pleasure in talking to people	17					0.425
**Percentage of variances explained**		31.90	10.57	8.95	7.62	6.97

#### SNS retest results (Table 3)

None of the items of the SNS scale was removed. The PCA for the SNS scale was also run over the whole sample (Total *n* = 206). The SNS scale items converged over a solution of five factors that had an Eigenvalue over 1, explaining a total of 61.95% of the variance. A Kaiser-Meyer-Olkin measure of sampling adequacy of 0.804 was found, with a significant Bartlett’s test of sphericity (*p* < 0.001) (Table 3).

**Table 3:** Results of the Varimax rotated matrix of the SNS items (Retest)

Factor	Item number	Alogia	Avolition	Social withdrawal	Anhedonia	Diminished emotional range
People often say that I don’t talk much	11	0.829				
I don’t have as much to talk about as most people	9	0.794				
I find it 10 times harder to talk than most people do	10	0.789				
With friends and family, I want to talk about things but it doesn’t come out	12	0.562				
It’s hard to stick to doing things on an everyday regular basis	14		0.865			
I know there are things I must do (get up or wash myself for example) but I have no energy	16		0.839			
There are many things I don’t do through lack of motivation or because I don’t feel like it	15		0.826			
I find it difficult to meet the objectives I set myself	13		0.464			
I’m better off alone, because I feel uncomfortable when anyone is near me	2			0.823		
I don’t particularly try to contact and meet friends (letters, telephone, text messaging, etc.)	4			0.720		
I prefer to be alone in my corner	1			0.700		
I’m not interested in going out with friends or family	3			0.697		
When I imagine doing one thing or another, I don’t feel any particular pleasure in the idea	19				0.912	
I find it hard to take pleasure even when doing things I have chosen to do	18				0.889	
I don’t take any great pleasure in talking to people	17				0.456	
I am not interested in having sex	20				0.410	
People say I’m not sad or happy and that I’m not often angry	5					0.687
It is difficult for people to know how I feel	8					0.676
Watching a sad or happy film, reading or listening to a sad or happy story does not especially make me want to cry or laugh	7					0.421
There are many happy or sad things in life but I don’t feel concerned by them	6					0.421
**Percentage of variances explained**		29.26	11.19	10.83	9.36	6.96

### Cronbach’s alpha values and Intraclass correlation coefficients between the test and retest

The Cronbach’s alpha values for the test and retest were very good (0.879 and 0.867 respectively). The ICC for the total SNS score was 0.569 (95% CI 0.433–0.673), 0.369 for factor 1 (95% CI 0.169–0.520), 0.423 for factor 2 (95% CI 0.240–0.561), 0.116 for factor 3 (95% CI -0.163-0.328), 0.345 for factor 4 (95% CI 0.138–0.502), and 0.391 for factor 5 (95% CI 0.199–0.537).

### Description of the SNS scale and subscales scores

The mean total SNS score was 17.33 ± 8.43 for the “test”, and 16.35 ± 7.50 for the “retest”. The average SNS sub-scores were SNS social withdrawal (3.12 ± 2.61), SNS decreased emotional feeling (3.85 ± 2.59), SNS alogia (4.91 ± 2.65), SNS abolition (4.28 ± 2.64), and SNS anhedonia (1.17 ± 1.48) (results of the test assay).

The mean PANSS total score was 77.51 ± 30.48 (median = 71), whereas the mean of the negative subscale score was 18.56 ± 8.04 (median = 18).

### Correlation between SNS and negative symptoms evaluated by the PANSS

The correlation coefficients between the SNS total score and the PANSS scale and subscales were as follows: total PANSS (r = 0.044; *p* = 0.530), positive PANSS score (r = − 0.106; *p* = 0.131), negative PANSS score (r = 0.204; *p* = 0.003), and general psychopathological PANSS score (r = 0.03; *p* = 0.530).

## Discussion

Negative symptoms in schizophrenia are of great importance for the patients and their families as they represent an unmet treatment need [6]. Choosing wisely the scales that allow us to correctly assess these symptoms would help clinicians develop an adequate treatment strategy [6]. Therefore, it was deemed essential to validate the Arabic version of the SNS and compare its results with those of the negative symptoms subscale of the PANSS in Lebanese patients with schizophrenia.

None of the items were removed from both factor analyses; the results described the five negative symptoms found in the literature. The Cronbach’s alpha values were similar to those of the French [12], Spanish [19] and Polish [20] versions. The ICC between the test and the retest was moderate in this study; this might be due to the fact that Lebanese patients had a more chronic disease, with a longer duration of illness, and were taking a higher chlorpromazine-equivalent dose compared to that in the original article of the SNS validation [10]. The results of the validation of the Arabic version are promising because the SNS is self-administered, unlike scales like the PANSS that require multiple trainings and in-depth expertise from healthcare professionals [21]. The validation steps [22] showed that the SNS is a valid tool for the self-assessment of negative symptoms in Lebanese patients with schizophrenia. In addition, the convergent and discriminant validity was confirmed since the SNS total score was correlated with the PANSS negative score only and not with other PANSS scores; this consolidates findings of a previous study [11], showing that the SNS is a valid tool for screening negative symptoms in clinical practice.

The weak correlation in our study between the negative PANSS subscale and the SNS scores confirms the previous results stating that the PANSS does not allow a good assessment of negative symptoms [21, 23]. Moreover, this lack of correlation between hetero-assessments tools and self-assessment ones are probably due to the fact that they reflect negative symptoms from two different perspectives: the clinician and the patient.

The mean SNS score in our sample was 17.33 and 16.35 in both tests respectively, higher than the means found in the French validation studies (16.6 and 14.9 respectively) [12] and in the Polish study (16.4 ± 9.7) [20], suggesting more severe negative symptoms among Lebanese patients. This higher level might be due to the fact that the Lebanese patients enrolled in this study had more severe symptoms since they were recruited from a specialized psychiatric hospital in Lebanon compared to patients from other studies, some of whom were recruited from clinics. Moreover, Lebanese patients might have a particular genetic background since 21.4% of them had a family history of schizophrenic episodes and 10.2% of other psychiatric disorders.

### Limitations and strengths

Several limitations could be noted in our study. For example, other scales, meeting the consensus guidelines on the agreement of negative symptoms [24, 25], could have been used to compare the results obtained with the SNS. Indeed, the negative PANSS subscale might not be the best scale to evaluate negative symptoms [21, 23], however, it was selected because it is the only scale validated in Arabic, and it has good validity and reliability [21]. Moreover, we did not apply the blinded self-assessment which might affect the clinicians’ ratings for the PANSS ratings. Another limitation related to the demographic characteristics of our population could be noted since more than 50% of patients included in this study were female and were older than those evaluated in other SNS validation articles [12, 26]. Therefore, our results may not be generalized to the whole population of Lebanese schizophrenic patients. Moreover, we only included chronic patients with a hospitalization of more than a year so that we could guarantee that they would still be at the hospital after 6 weeks for the retest. Finally, the level of insight was not assessed in the present study, but previous data showed no correlation between the level of insight and the SNS scores [10]. Nevertheless, despite all these limitations, and to the best of our knowledge, this is first test-retest study for the validation of the SNS that enrolls a large number of randomly selected patients.

## Conclusion

Our study validated the SNS tool and confirmed its validity and reliability in Lebanese patients with schizophrenia. Using this scale would help improve treatment by optimizing and individualizing the diagnosis of these symptoms, thereby reducing the risk of misdiagnosis and treatment errors, and reducing the negative impact of resistance to treatment.

## Data Availability

The authors do not have the right to share any data information as per their institutions policies.

## Abbreviations

PANSS: Positive and Negative Syndrome Scale
SNS: Self-evaluation of negative symptoms scale
HPC: Psychiatric Hospital of the Cross
